# Correlation between *ESR1* and *APOE* gene polymorphisms and risk of osteonecrosis of the femoral head: a case–control study

**DOI:** 10.1186/s13018-023-04447-4

**Published:** 2023-12-15

**Authors:** Yuan Wang, Xiaoya Ma, Jinping Guo, Yujie Li, Yuyan Xiong

**Affiliations:** 1 College of Life Sciences, Northwest University, Xi’an, 710069 Shaanxi China; 2 Provincial Key Laboratory of Biotechnology of Shaanxi, Northwest University, Xi’an, 710069 Shaanxi China; 3 Key Laboratory of Resource Biology and Biotechnology in Western China, Ministry of Education, School of Life Sciences, Northwest University, Xi’an, 710069 Shaanxi China; 4 Department of Joint Surgery, Affiliated Hospital of Weifang Medical University, Weifang, 261031 Shandong China

**Keywords:** *ESR1*, *APOE*, Osteonecrosis of the femoral head, Gene polymorphisms, Risk

## Abstract

**Background:**

Osteonecrosis of the femoral head (ONFH) is a disease with a high disability rate, and genetic factors are closely related to its pathogenesis. This study aimed to investigate the possible correlation between *ESR1* and *APOE* gene polymorphisms and the risk of ONFH.

**Methods:**

In this case–control study, the potential association between three genetic variants (rs2982573 C < T, rs10872678 C < T, and rs9322332 A < C) of the *ESR1* gene and two genetic variants (rs7259620 A < G and rs769446 C < T) of the *APOE* gene with the risk of ONFH was investigated. Correlations between gene polymorphisms and ONFH risk were assessed using logistic regression analysis, with calculation of odds ratios (ORs) and 95% confidence intervals (CIs).

**Results:**

The overall analysis demonstrated that rs9322332 in the *ESR1* gene exhibited a correlation with a decreased risk of ONFH under the homozygous (AA vs.CC: OR = 0.69, 95% CI [0.53–0.90], *p* = 0.006), dominant (CA + AA vs. CC: OR = 0.70, 95% CI [0.54–0.90], *p* = 0.006), and additive (OR = 0.79, 95% CI [0.66–0.95], *p* = 0.013) models. The stratification analysis revealed that rs9322332 was linked to a lower risk of ONFH in subgroups characterized by individuals aged over 51 years and non-smokers. Nevertheless, there were no notable correlations found between *ESR1* rs2982573 and rs10872678, as well as *APOE* rs7259620 and rs769446, with the risk of ONFH.

**Conclusion:**

*ESR1*-rs9322332 is closely linked to a decreased risk of ONFH, thereby enhancing our understanding of the relationship between gene polymorphisms and ONFH.

**Supplementary Information:**

The online version contains supplementary material available at 10.1186/s13018-023-04447-4.

## Introduction

Osteonecrosis of the femoral head (ONFH), also referred to as ischemic necrosis of the femoral head, is a frequently seen and challenging condition in orthopedics. It is characterized by the interruption or impairment of blood supply to the femoral head, leading to the death and insufficient repair of bone cells and bone marrow cells. Subsequently, this causes structural changes in the femoral head and its eventual collapse, resulting in joint pain and dysfunction [1]. According to a large-scale epidemiological survey, it was found that the estimated cumulative number of non-traumatic ONFH patients in China has reached 8.12 million, with males showing a significantly higher prevalence compared to females, and urban areas having a higher prevalence than rural areas [2]. Nowadays, various methods have been used in the clinical treatment of ONFH, such as osteotomies [3], total hip arthroplasty (THA) [4], and bone marrow-derived cell therapies (BMCTs) combined with core decompression (CD) [5], but their effectiveness and safety are limited. A comprehensive elucidation of the pathogenesis of ONFH can provide important directions for its precise treatment. Previous studies have revealed that multiple factors, including single nucleotide polymorphisms (SNPs), may be closely associated with its occurrence [6–8]. Hence, the investigation of the influence of SNPs on the risk of ONFH holds great significance in terms of preventing, diagnosing, and effectively treating this condition.

Estrogen receptor (ER) is a group of nuclear receptor superfamily members that function as transcription factors in the nucleus, consisting of two types: ERα and ERβ [9]. The Estrogen Receptor 1 (*ESR1*) gene encodes the ERα, which is localized within the bone and plays a crucial role in regulating bone metabolism [10]. Relevant studies have shown that estrogen can promote osteoblast proliferation and inhibit osteoclast activity by binding with *ESR1*, thus playing a physiological role, while estrogen deficiency may cause changes in bone microstructure, resulting in increased cortical bone vascular aperture, increased bone trabecular separation and decreased bone trabecular number, thus leading to the occurrence of ONFH [11–13]. Currently, there has been research conducted to investigate the impact of *ESR1* polymorphisms on the quality of the femoral head in patients with Turner syndrome, revealing that *ESR1* rs2234693 is potentially linked to decreased bone mineral density (BMD) in the femoral neck and total hip regions [14]. However, the available research on the association between *ESR1* gene polymorphisms and the risk of ONFH is scarce.

Apolipoprotein E (*APOE*), located on chromosome 19, plays a crucial role in plasma lipid metabolism by facilitating the hepatocyte-mediated uptake and removal of chylomicrons, very low-density lipoproteins (VLDL), and high-density lipoproteins (HDL) lipoproteins [15]. It is reported that *APOE*-deficient individuals may exhibit severe hyperlipidemia, which can potentially influence the microcirculation of the femoral head and contribute to the development of ONFH [16, 17]. At present, there is a dearth of research investigating the potential link between *APOE* gene polymorphisms and the risk of developing steroid-induced ONFH. Some studies have suggested a possible association between the rs7412 C/T and rs429358 T/C loci and an elevated risk of SONFH [18]. Nevertheless, as of now, there is still no reported evidence regarding the specific relationship between *APOE* gene polymorphisms and ONFH.

Given that *ESR1* and *APOE* gene polymorphisms may be linked to the ONFH, this study seeks to assess the influence of *ESR1* gene (rs2982573 C < T, rs10872678 C < T and rs9322332 A < C) and *APOE* gene (rs7259620 A < G and rs769446 C < T) polymorphisms on the susceptibility to ONFH. The findings of this study are anticipated to shed light on potential biomarkers for the diagnosis and treatment of ONFH.

## Materials and methods

### Study participants

This study included a sample of 505 individuals diagnosed with ONFH and 512 healthy controls, all of whom were obtained from the Affiliated Hospital of Weifang Medical University and the Second Affiliated Hospital of Inner Mongolia Medical University. The inclusion criteria for ONFH cases were as follows: (1) Patients experiencing pain in the hip joint, buttock, or groin area, accompanied by pain in the knee joint and restricted internal and external rotation of the hip joint; (2) Diagnosis of ONFH confirmed by X-ray, computed tomography (CT), and magnetic resonance imaging (MRI); (3) No history of direct trauma, osteoarthritis, ankylosing spondylitis, hip joint-related diseases (e.g., hip joint synovitis), cardiovascular and cerebrovascular diseases, metabolic disorders, or bone metastasis. The group of healthy controls consisted of individuals who met the following inclusion criteria: (1) No pain in the hip joint, buttock, or groin area; (2) No evidence of lesions on imaging examinations; (3) No chronic alcohol use or steroid use; (4) No history of direct trauma, osteoarthritis, ankylosing spondylitis, cardiovascular and cerebrovascular diseases, metabolic disorders, or bone metastasis. Demographic characteristics and clinical data of the study participants were collected through questionnaires and a review of patient medical records. Additionally, this study was approved by the ethics committee of the Affiliated Hospital of Weifang Medical University and the ethics committee of the Second Affiliated Hospital of Inner Mongolia Medical University, and conducted in compliance with the principles outlined in the Declaration of Helsinki. Informed consent was obtained from all participants before the commencement of the experiment.

### DNA extraction and genotyping

Three SNPs (rs2982573, rs10872678, and rs9322332) in the *ESR1* gene, as well as two SNPs (rs7259620 and rs769446) in the *APOE* gene, were selected for genotyping. These SNPs were selected based on the following process: (1) Data on *ESR1* and *APOE* gene polymorphisms were obtained from the 1000 Genomes Project database; (2) Screening criteria were implemented, necessitating a minor allele frequency (MAF) above 5% and Hardy–Weinberg equilibrium (HWE) exceeding 0.01; (3) The SNPs to be studied were identified through a combination of primer design and an extensive literature search. After a 12-h fasting period, 5 mL of peripheral venous blood was collected from the study participants using a vacuum blood collection tube containing EDTA-K2 anticoagulant, and mixed upside down and used in subsequent experiments. Genomic DNA extraction was performed using a genomic DNA isolation kit (GoldMag Biotechnology) following the manufacturer’s instructions. The concentration of the DNA was measured using a Nanodrop 2000 spectrophotometer (Thermo, USA). SNP genotyping was carried out utilizing the Agena MassARRAY platform (Agena Bioscience, USA), and the data analysis was performed using the Agena Typer 4.0 software. Additional file 1: Table S1 contains the listed sequences of the primers.

### Statistical analysis

**Fig. 1:**
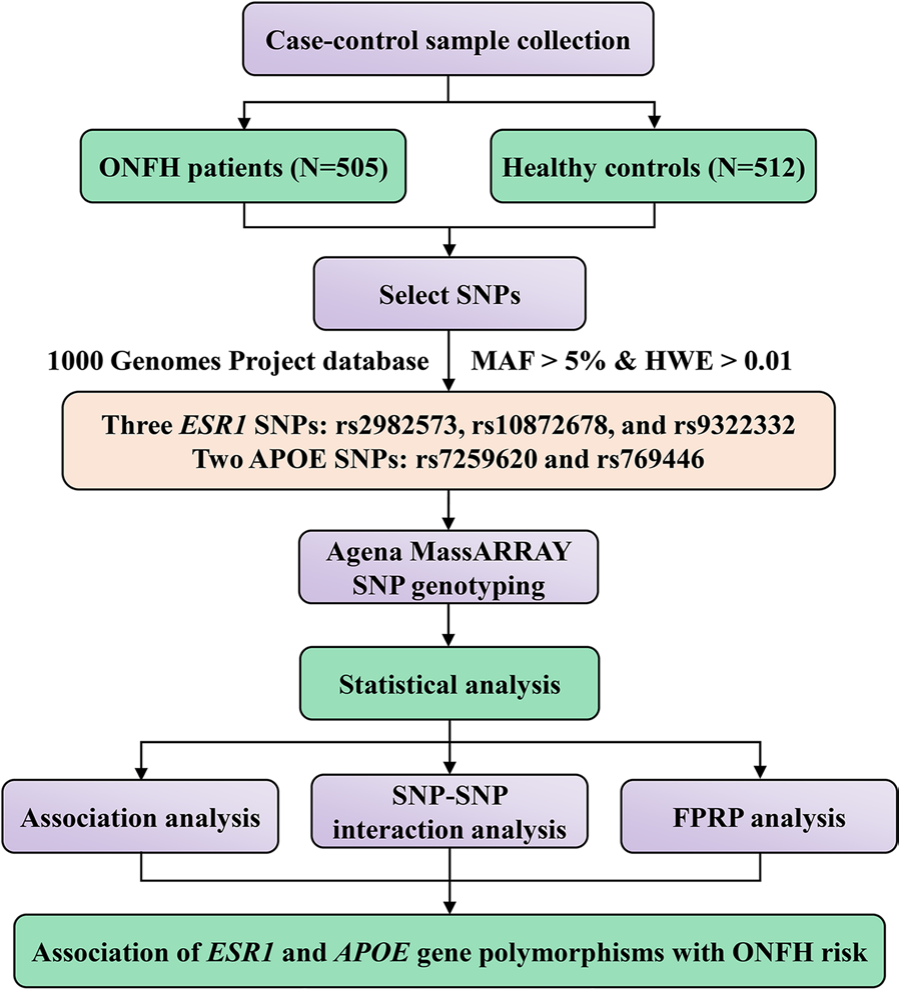
Flowchart illustrating the analysis of the association between *ESR1* and *APOE* gene polymorphisms and ONFH

The characteristics of the study participants were analyzed using the *t*-test for continuous variables and χ^2^ test for categorical variables. HWE in controls was calculated using the χ^2^ test to further explain the good representativeness of the study population. Logistic regression analysis with odds ratios (ORs) and corresponding 95% confidence intervals (CIs) was used to assess the association between *ESR1* and *APOE* gene polymorphisms and the risk of ONFH. Multifactor dimensionality reduction (MDR) analysis was employed to explore SNP-SNP interactions. The reliability of significant findings was assessed using false positive report probability (FPRP) analysis. Statistical significance was set at a *p* value of less than 0.05. The flowchart of this study is shown in Fig. 1.

## Results

### Demographic characteristics of the study participants

This case–control study encompassed a total of 1,017 participants, with 505 cases of ONFH (284 males and 221 females) and 512 healthy controls (305 males and 207 females). The average age of ONFH patients was 51.65 ± 14.43, whereas the average age of healthy controls was 50.59 ± 14.39. It is noteworthy that a statistically significant difference in smoking (*p* = 0.004) factor was observed between the cases and controls, whereas there were no significant differences in terms of age (*p* = 0.242) and gender (*p* = 0.282) distribution between the two groups. The detailed characteristics of the study participants are shown in Table 1.

**Table 1:** Basic characteristics of study participants

Parameter	Case (n = 505)	Control (n = 512)	*p*
Age (years, Mean ± SD)	51.65 ± 14.43	50.59 ± 14.39	0.242^a^
> 51	281 (55.6%)	247 (48.2%)	
≤ 51	224 (44.4%)	265 (51.8%)	
Gender, n (%)			0.282^b^
Male	284 (56.2%)	305 (59.6%)	
Female	221 (43.8%)	207 (40.4%)	
Smoking			**0.004**^b^
Yes	227 (45.0%)	276 (53.9%)	
No	278 (55.0%)	236 (46.1%)	
Clinical staging			
III–IV	199 (39.4%)	–	–
I–II	69 (13.7%)	–	–
Missing data	237 (46.9%)	–	**–**

SD: standard deviation

*p*^a^ value was obtained by t-test

*p*^b^ value was obtained by χ2 test

Bold values indicate statistical significance

### Association of *ESR1* and *APOE* allele frequencies with ONFH risk

The basic information and MAF of SNPs for *ESR1* and *APOE* are provided in Table 2. It is worth noting that all five SNPs in the control group followed HWE. Through allele model analysis, a χ^2^ test identified one SNP that exhibited a close association with ONFH. Specifically, the A allele of rs9322332 was found to be significantly associated with a reduced risk of ONFH, showing a 0.81-fold decrease (OR = 0.81, 95% CI [0.68–0.97], *p* = 0.020).

**Table 2:** Basic information and allele frequencies of rs2982573, rs10872678, rs9322332, rs7259620, and rs769446

SNP_ID	Gene	Chr	Base pair	Allele	MAF		HWE	OR (95% CI)	χ2	*p*
					Case	Controls	*p*			
rs2982573	*ESR1*	6	151,689,399	C < T	0.156	0.156	0.738	1.01 (0.79–1.28)	0.003	0.958
rs10872678	*ESR1*	6	151,718,829	C < T	0.220	0.219	0.195	1.01 (0.82–1.24)	0.005	0.944
rs9322332	*ESR1*	6	151,845,666	A < C	0.371	0.422	0.057	0.81 (0.68–0.97)	5.399	**0.020**
rs7259620	*APOE*	19	44,904,531	A < G	0.298	0.294	0.524	1.02 (0.84–1.23)	0.033	0.856
rs769446	*APOE*	19	44,905,371	C < T	0.084	0.096	0.607	0.87 (0.64–1.18)	0.828	0.363

SNP: single nucleotide polymorphism; Chr: chromosome; MAF: minor allele frequency; HWE: Hardy–Weinberg equilibrium; OR: odds ratio; 95% CI: 95% confidence interval; *χ*^2^: chi-square

Bold values indicate statistical significance

*p* value was obtained by *χ*^2^ test

### Overall analysis of the association of *ESR1* and *APOE* gene polymorphisms with ONFH risk

**Fig. 2:**
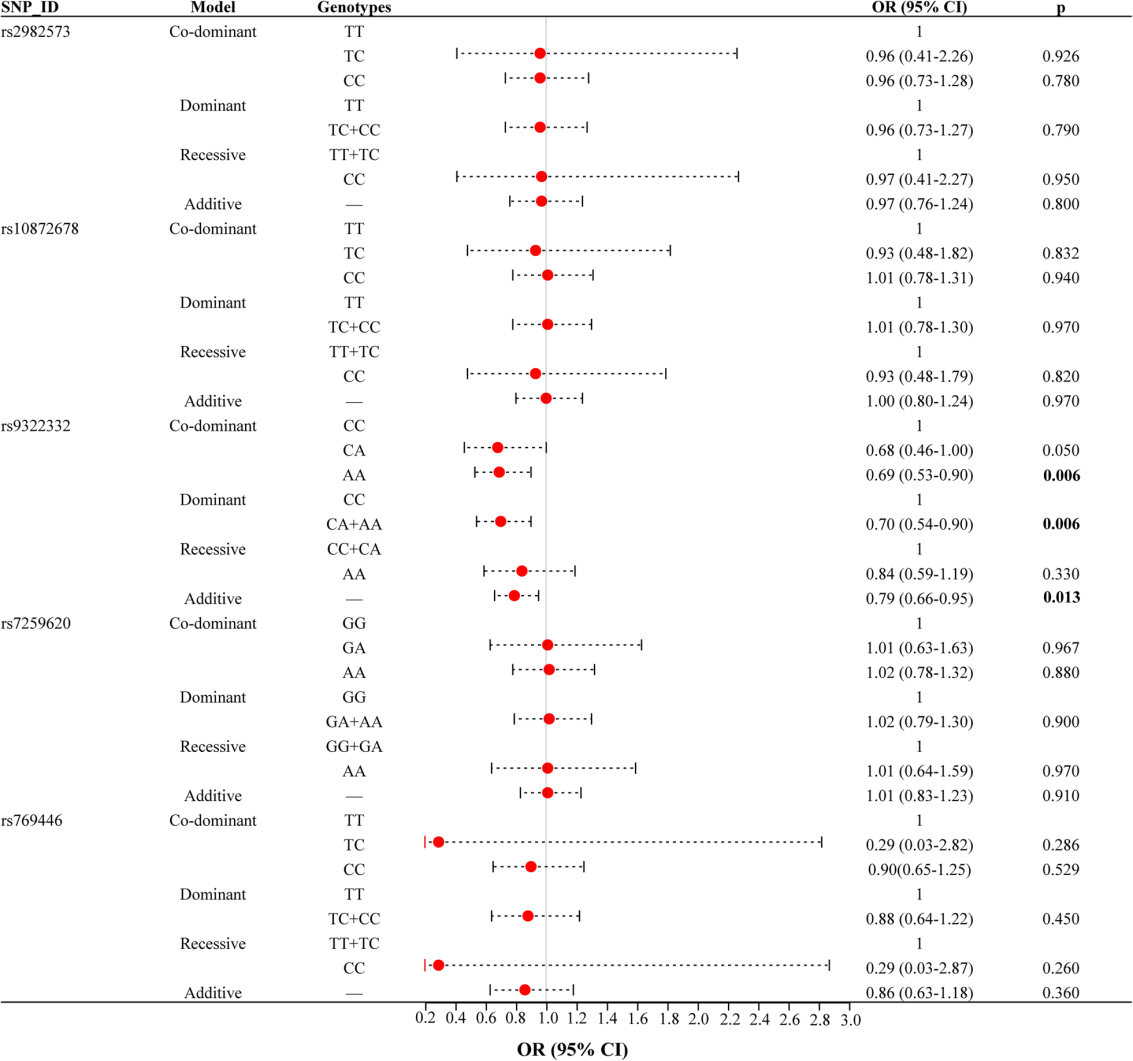
Forest maps of associations between *ESR1* and *APOE* gene polymorphisms and ONFH in different genetic models

This study investigated the correlation between *ESR1* and *APOE* gene polymorphisms and the risk of ONFH using different genetic models, including co-dominant, dominant, recessive, and additive models. The findings of the overall analysis examining the association of *ESR1* and *APOE* gene polymorphisms with ONFH risk are summarized in Fig. 2 and Table 3. The findings indicated a significant association between *ESR1*-rs9322332 and a decreased risk of ONFH in the overall analysis, especially in homozygous (OR = 0.69, 95% CI [0.53–0.90], *p* = 0.006), as well as in dominant (OR = 0.70, 95% CI [0.54–0.90], *p* = 0.006) and additive (OR = 0.79, 95% CI [0.66–0.95], *p* = 0.013) models. However, this study found no association between other SNPs of *ESR1* and *APOE* genes and ONFH risk.

**Table 3:** Association between *ESR1* and *APOE* gene polymorphisms and ONFH risk

SNP_ID	Model	Genotypes	Case	Controls	Adjusted by age, gender, and smoking	
					OR (95% CI)	*p*
rs2982573	Co-dominant	TT	358	363	1	
		TC	136	137	0.96 (0.41–2.26)	0.926
		CC	11	11	0.96 (0.73–1.28)	0.780
	Dominant	TT	358	363	1	
		TC + CC	147	148	0.96 (0.73–1.27)	0.790
	Recessive	TT + TC	494	500	1	
		CC	11	11	0.97 (0.41–2.27)	0.950
	Additive	–	–	–	0.97 (0.76–1.24)	0.800
rs10872678	Co-dominant	TT	358	305	1	
		TC	186	184	0.93 (0.48–1.82)	0.832
		CC	18	19	1.01 (0.78–1.31)	0.940
	Dominant	TT	301	305	1	
		TC + CC	204	203	1.01 (0.78–1.30)	0.970
	Recessive	TT + TC	487	489	1	
		CC	18	19	0.93 (0.48–1.79)	0.820
	Additive	–	–	–	1.00 (0.80–1.24)	0.970
rs9322332	Co-dominant	CC	200	160	1	
		CA	235	271	0.68 (0.46–1.00)	0.050
		AA	70	80	0.69 (0.53–0.90)	**0.006**
	Dominant	CC	200	160	1	
		CA + AA	305	351	0.70 (0.54–0.90)	**0.006**
	Recessive	CC + CA	435	431	1	
		AA	70	80	0.84 (0.59–1.19)	0.330
	Additive	–	–	–	0.79 (0.66–0.95)	**0.013**
rs7259620	Co-dominant	GG	245	252	1	
		GA	218	219	1.01 (0.63–1.63)	0.967
		AA	41	41	1.02 (0.78–1.32)	0.880
	Dominant	GG	245	252	1	
		GA + AA	259	260	1.02 (0.79–1.30)	0.900
	Recessive	GG + GA	463	471	1	
		AA	41	41	1.01 (0.64–1.59)	0.970
	Additive	–	–	–	1.01 (0.83–1.23)	0.910
rs769446	Co-dominant	TT	421	417	1	
		TC	83	92	0.29 (0.03–2.82)	0.286
		CC	1	3	0.90(0.65–1.25)	0.529
	Dominant	TT	421	417	1	
		TC + CC	84	95	0.88 (0.64–1.22)	0.450
	Recessive	TT + TC	504	509	1	
		CC	1	3	0.29 (0.03–2.87)	0.260
	Additive	–	–	–	0.86 (0.63–1.18)	0.360

ONFH: Osteonecrosis of the femoral head; SNP: single nucleotide polymorphism; OR: odds ratio; 95% CI: 95% confidence interval

*p* value was obtained by logistic regression analysis with adjustments for age, gender, and smoking

Bold values indicate statistical significance

### Stratified analysis of the association of *ESR1* and *APOE* gene polymorphisms with ONFH risk

To further investigate the correlation of *ESR1* and *APOE* gene polymorphisms with ONFH risk, we performed stratified analyses based on age (Table 4), gender (Table 5), smoking status (Table 6), and clinical staging (Additional file 1: Table S2). Under different genetic models, the association between rs9322332 and a reduced risk of ONFH was observed in specific subgroups, including individuals older than 51 years (CA vs CC: OR = 0.47, 95% CI [0.28–0.80], *p* = 0.005; AA vs CC: OR = 0.52, 95% CI [0.35–0.77], *p* = 0.001; CA + AA vs CC: OR = 0.51, 95% CI [0.35–0.74], *p* < 0.001; Additive: OR = 0.65, 95% CI [0.50–0.84], *p* = 0.001), females (AA vs CC: OR = 0.51, 95% CI [0.34–0.78], *p* = 0.002; CA + AA vs CC: OR = 0.55, 95% CI [0.37–0.82], *p* = 0.004), and non-smokers (CA + AA vs CC: OR = 0.68, 95% CI [0.47–0.99], *p* = 0.042; Additive: OR = 0.77, 95% CI [0.59–0.99], *p* = 0.042). However, this study did not observe any associations between several other SNPs (rs2982573, rs10872678, rs7259620, and rs769446) and ONFH when analyzed using different genetic models.

**Table 4:** Association between *ESR1* and *APOE* gene polymorphisms and ONFH risk stratified by age

SNP_ID	Model	Genotypes	Age˃51				Age ≤ 51			
			Case	Controls	OR (95% CI)	*p*	Case	Controls	OR (95% CI)	*p*
rs2982573	Co-dominant	TT	194	167	1		164	196	1	
		TC	79	73	0.99 (0.35–2.81)	0.990	57	64	0.91 (0.20–4.12)	0.900
		CC	8	7	0.94 (0.64–1.38)	0.753	3	4	1.07 (0.71–1.61)	0.760
	Dominant	TT	194	167	1		164	196	1	
		TC + CC	87	80	0.95 (0.65–1.37)	0.765	60	68	1.06 (0.71–1.59)	0.788
	Recessive	TT + TC	273	240	1		221	260	1	
		CC	8	7	1.01 (0.36–2.84)	0.983	3	4	0.89 (0.20–4.04)	0.883
	Additive	–	–	–	0.96 (0.69–1.32)	0.798	–	–	1.04 (0.72–1.51)	0.833
rs10872678	Co-dominant	TT	169	147	1		132	158	1	
		TC	98	88	1.32 (0.55–3.15)	0.532	88	96	1.06 (0.60–1.87)	0.843
		CC	14	9	1.00 (0.69–1.44)	0.986	4	10	0.89 (0.61–1.32)	0.573
	Dominant	TT	169	147	1		132	158	1	
		TC + CC	112	97	1.03 (0.72–1.46)	0.878	92	106	0.93 (0.64–1.34)	0.689
	Recessive	TT + TC	267	235	1		220	254	1	
		CC	14	9	1.32 (0.56–3.13)	0.526	4	10	1.13 (0.67–1.90)	0.645
	Additive	–	–	–	1.06 (0.78–1.42)	0.723	–	–	0.99 (0.76–1.30)	0.959
rs9322332	Co-dominant	CC	116	65	1		84	95	1	
		CA	127	135	0.47 (0.28–0.80)	**0.005**	108	136	0.48 (0.15–1.58)	0.229
		AA	38	46	0.52 (0.35–0.77)	**0.001**	32	34	1.10 (0.76–1.60)	0.606
	Dominant	CC	116	65	1		84	95	1	
		CA + AA	165	181	0.51 (0.35–0.74)	**< 0.001**	140	170	1.05 (0.73–1.50)	0.814
	Recessive	CC + CA	243	200	1		192	231	1	
		AA	38	46	0.69 (0.43–1.11)	0.127	32	34	0.47 (0.14–1.51)	0.202
	Additive	–	–	–	0.65 (0.50–0.84)	**0.001**	–	–	0.97 (0.70–1.34)	0.853
rs7259620	Co-dominant	GG	137	115	1		108	137	1	
		GA	117	111	1.03 (0.55–1.93)	0.931	101	108	0.96 (0.47–1.98)	0.920
		AA	26	21	0.88 (0.61–1.26)	0.475	15	20	1.18 (0.82–1.72)	0.373
	Dominant	GG	137	115	1		108	137	1	
		GA + AA	143	132	0.90 (0.64–1.27)	0.553	116	128	1.15 (0.81–1.64)	0.443
	Recessive	GG + GA	254	226	1		209	245	1	
		AA	26	21	1.10 (0.60–2.00)	0.769	15	20	0.89 (0.44–1.79)	0.749
	Additive	–	–	–	0.96 (0.73–1.25)	0.742	–	–	1.07 (0.81–1.43)	0.629
rs769446	Co-dominant	TT	232	200	1		189	217	1	
		TC	48	45	–	0.999	34	47	1.18 (0.07–19.12)	0.905
		CC	0	2	0.93 (0.59–1.45)	0.736	1	1	0.83 (0.51–1.35)	0.457
	Dominant	TT	232	200	1		189	217	1	
		TC + CC	49	47	0.88 (0.57–1.38)	0.588	35	48	0.84 (0.52–1.36)	0.474
	Recessive	TT + TC	271	245	1		223	264	1	
		CC	0	2	–	0.999	1	1	1.22 (0.08–19.72)	0.887
	Additive	–	–	–	0.84 (0.55–1.30)	0.442	–	–	0.85 (0.54–1.36)	0.505

ONFH: Osteonecrosis of the femoral head; SNP: single nucleotide polymorphism; OR: odds ratio; 95% CI: 95% confidence interval

*p* value was obtained by logistic regression analysis with adjustments for gender and smoking

Bold values indicate statistical significance

**Table 5:** Association between *ESR1* and *APOE* gene polymorphisms and ONFH risk stratified by gender

SNP_ID	Model	Genotypes	Male				Female			
			Case	Controls	OR (95% CI)	*p*	Case	Controls	OR (95% CI)	*p*
rs2982573	Co-dominant	TT	200	220	1		158	143	1	
		TC	78	75	0.72 (0.25–2.07)	0.543	58	62	2.23 (0.42–11.66)	0.344
		CC	6	9	1.14 (0.79–1.65)	0.491	5	2	0.84 (0.55–1.28)	0.411
	Dominant	TT	200	220	1		158	143	1	
		TC + CC	84	84	1.09 (0.76–1.57)	0.622	63	64	0.88 (0.58–1.34)	0.548
	Recessive	TT + TC	278	295	1		216	205	1	
		CC	6	9	0.70 (0.24–1.98)	0.498	5	2	2.34 (0.45–12.22)	0.312
	Additive	–	–	–	1.04 (0.76–1.42)	0.823	–	–	0.95 (0.65–1.39)	0.775
rs10872678	Co-dominant	TT	164	184	1		137	121	1	
		TC	112	106	0.59 (0.25–1.44)	0.250	74	78	2.20 (0.67–7.20)	0.193
		CC	8	15	1.18 (0.84–1.66)	0.331	10	4	0.83 (0.56–1.24)	0.372
	Dominant	TT	164	184	1		137	121	1	
		TC + CC	120	121	1.11 (0.80–1.54)	0.534	84	82	0.90 (0.61–1.33)	0.595
	Recessive	TT + TC	276	290	1		211	199	1	
		CC	8	15	0.56 (0.23–1.34)	0.190	10	4	2.35 (0.73–7.63)	0.154
	Additive	–	–	–	1.01 (0.76–1.34)	0.932	–	–	1.00 (0.71–1.41)	0.997
rs9322332	Co-dominant	CC	103	97	1		97	63	1	
		CA	142	154	0.68 (0.41–1.12)	0.128	93	117	0.74 (0.40–1.38)	0.347
		AA	39	54	0.87 (0.61–1.25)	0.446	31	26	0.51 (0.34–0.78)	**0.002**
	Dominant	CC	103	97	1		97	63	1	
		CA + AA	181	208	0.82 (0.58–1.15)	0.256	124	143	0.55 (0.37–0.82)	**0.004**
	Recessive	CC + CA	245	251	1		190	180	1	
		AA	39	54	0.74 (0.47–1.16)	0.186	31	26	1.10 (0.62–1.93)	0.746
	Additive	–	–	–	0.83 (0.66–1.06)	0.135	–	–	0.75 (0.56–1.00)	0.052
rs7259620	Co-dominant	GG	138	159	1		107	93	1	
		GA	124	125	1.20 (0.63–2.28)	0.578	94	94	0.81 (0.41–1.62)	0.551
		AA	22	21	1.15 (0.82–1.61)	0.430	19	20	0.86 (0.58–1.29)	0.474
	Dominant	GG	138	159	1		107	93	1	
		GA + AA	146	146	1.15 (0.83–1.59)	0.388	113	114	0.85 (0.58–1.25)	0.420
	Recessive	GG + GA	262	284	1		201	187	1	
		AA	22	21	1.13 (0.61–2.10)	0.705	19	20	0.87 (0.45–1.69)	0.682
	Additive	–	–	–	1.12 (0.86–1.45)	0.397	–	–	0.89 (0.66–1.19)	0.421
rs769446	Co-dominant	TT	246	250	1		175	167	1	
		TC	37	55	–	0.999	46	37	–	0.999
		CC	1	0	0.68 (0.43–1.07)	0.097	0	3	1.20 (0.74–1.95)	0.454
	Dominant	TT	246	250	1		175	167	1	
		TC + CC	38	55	0.70 (0.45–1.10)	0.120	46	40	1.11 (0.69–1.79)	0.663
	Recessive	TT + TC	283	305	1		221	204	1	
		CC	1	0	–	0.999	0	3	–	0.999
	Additive	–	–	–	0.73 (0.47–1.13)	0.157	–	–	1.01 (0.65–1.59)	0.954

ONFH: Osteonecrosis of the femoral head; SNP: single nucleotide polymorphism; OR: odds ratio; 95% CI: 95% confidence interval

*p* value was obtained by logistic regression analysis with adjustments for age and smoking

Bold values indicate statistical significance

**Table 6:** Association between *ESR1* and *APOE* gene polymorphisms and ONFH risk stratified by smoking status

SNP_ID	Model	Genotypes	Smoking-Yes				Smoking-No			
			Case	Controls	OR (95% CI)	*p*	Case	Controls	OR (95% CI)	*p*
rs2982573	Co-dominant	TT	178	198	1		180	165	1	
		TC	46	72	0.56 (0.14–2.29)	0.421	90	65	1.34 (0.42–4.19)	0.621
		CC	3	6	0.70 (0.46–1.07)	0.102	8	5	1.26 (0.86–1.85)	0.244
	Dominant	TT	178	198	1		180	165	1	
		TC + CC	49	78	0.69 (0.46–1.04)	0.079	98	70	1.26 (0.87–1.84)	0.223
	Recessive	TT + TC	224	270	1		270	230	1	
		CC	3	6	0.61 (0.15–2.48)	0.491	8	5	1.24 (0.40–3.88)	0.709
	Additive	–	–	–	0.71 (0.49–1.04)	0.077	–	–	1.23 (0.88–1.71)	0.232
rs10872678	Co-dominant	TT	147	161	1		154	144	1	
		TC	73	106	0.83 (0.30–2.31)	0.727	113	78	0.99 (0.41–2.42)	0.989
		CC	7	9	0.76 (0.52–1.10)	0.147	11	10	1.35 (0.93–1.95)	0.114
	Dominant	TT	147	161	1		154	144	1	
		TC + CC	80	115	0.76 (0.53–1.10)	0.148	124	88	1.31 (0.91–1.87)	0.143
	Recessive	TT + TC	220	267	1		267	222	1	
		CC	7	9	0.92 (0.34–2.53)	0.876	11	10	0.88 (0.37–2.13)	0.785
	Additive	–	–	–	0.81 (0.58–1.11)	0.187	–	–	1.20 (0.88–1.63)	0.245
rs9322332	Co-dominant	CC	91	88	1		109	72	1	
		CA	107	150	0.74 (0.42–1.31)	0.306	128	121	0.61 (0.36–1.04)	0.069
		AA	29	38	0.70 (0.48–1.03)	0.070	41	42	0.71 (0.48–1.04)	0.081
	Dominant	CC	91	88	1		109	72	1	
		CA + AA	136	188	0.71 (0.49–1.03)	0.067	169	163	0.68 (0.47–0.99)	**0.042**
	Recessive	CC + CA	198	238	1		237	193	1	
		AA	29	38	0.92 (0.54–1.54)	0.743	41	42	0.75 (0.47–1.21)	0.234
	Additive	–	–	–	0.82 (0.62–1.07)	0.137	–	–	0.77 (0.59–0.99)	**0.042**
rs7259620	Co-dominant	GG	119	130	1		126	122	1	
		GA	89	123	0.87 (0.45–1.68)	0.676	129	96	1.16 (0.59–2.27)	0.672
		AA	19	23	0.80 (0.55–1.16)	0.236	22	18	1.33 (0.92–1.92)	0.128
	Dominant	GG	119	130	1		126	122	1	
		GA + AA	108	146	0.81 (0.57–1.15)	0.243	151	114	1.30 (0.92–1.85)	0.142
	Recessive	GG + GA	208	253	1		255	218	1	
		AA	19	23	0.96 (0.51–1.82)	0.904	22	18	1.01 (0.53–1.94)	0.972
	Additive	–	–	–	0.87 (0.66–1.15)	0.335	–	–	1.18 (0.89–1.56)	0.239
rs769446	Co-dominant	TT	186	223	1		235	194	1	
		TC	40	53	–	0.999	43	39	–	0.999
		CC	1	0	0.88 (0.56–1.39)	0.586	0	3	0.92 (0.57–1.48)	0.735
	Dominant	TT	186	223	1		235	194	1	
		TC + CC	41	53	0.91 (0.57–1.43)	0.667	43	42	0.86 (0.54–1.37)	0.520
	Recessive	TT + TC	226	276	1		278	233	1	
		CC	1	0	–	0.999	0	3	–	0.999
	Additive	–	–	–	0.94 (0.60–1.46)	0.771	–	–	0.80 (0.51–1.25)	0.332

ONFH: Osteonecrosis of the femoral head; SNP: single nucleotide polymorphism; OR: odds ratio; 95% CI: 95% confidence interval

*p* value was obtained by logistic regression analysis with adjustments for age and gender

Bold values indicate statistical significance

### SNP-SNP interaction analysis based on MDR analysis

**Fig. 3:**
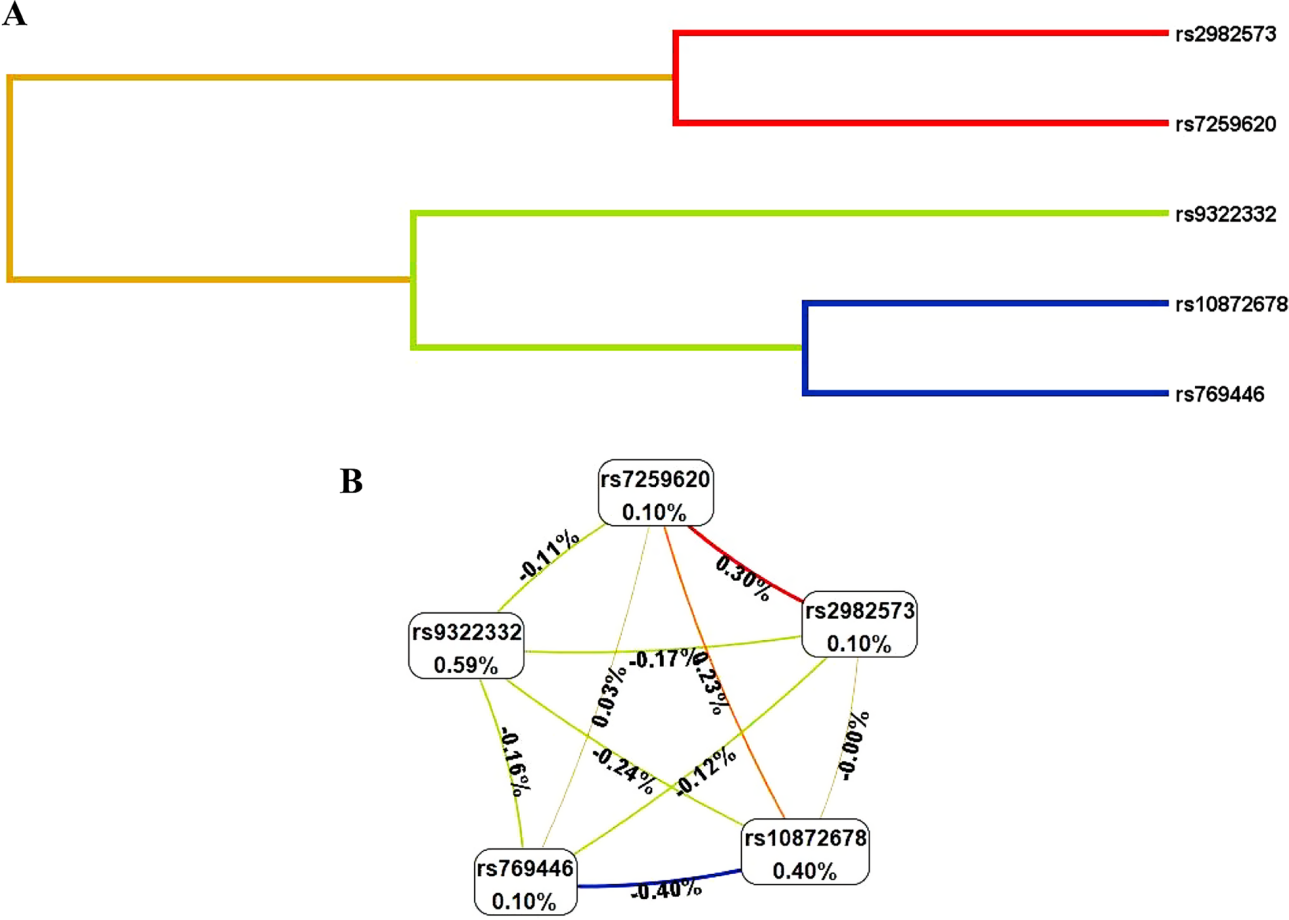
Dendrogram **A** and circle graph **B** of SNP-SNP interaction among *ESR1* and *APOE* gene polymorphisms by MDR method

MDR software was used to analyze SNP-SNP interactions among *ESR1* and *APOE* gene polymorphisms (Table 7). Consequently, the most effective single-locus prediction model identified was rs9322332, achieving a cross-validation consistency (CVC) of 10/10 and a testing balanced accuracy of 0.539 (*p* = 0.009). Furthermore, the optimal multi-locus prediction model was a combination of five loci (rs2982573, rs10872678, rs9322332, rs7259620, and rs769446), demonstrating a CVC of 10/10 and a testing balanced accuracy of 0.551 (*p* < 0.001). Additionally, the interaction between each locus was demonstrated in the dendrogram (Fig. 3A) and the circle graph (Fig. 3B). In Fig. 3B, the most significant interaction was observed between rs2982573 and rs7259620, with an information gain (IG) value of 0.30%.

**Table 7:** SNP-SNP interaction models of candidate SNPs analyzed by the MDR method

Model	Bal. Acc. CV training	Bal. Acc. CV testing	CVC	*p*
rs9322332	0.540	0.539	10/10	**0.009**
rs10872678/rs9322332	0.554	0.511	6/10	**0.001**
rs10872678/rs9322332/rs7259620	0.585	0.554	9/10	** < 0.001**
rs10872678/rs9322332/rs7259620/rs769446	0.605	0.551	9/10	** < 0.001**
rs2982573/rs10872678/rs9322332/rs7259620/rs769446	0.623	0.551	10/10	** < 0.001**

*SNP* single nucleotide polymorphism; *MDR* multifactor dimensionality reduction; *Bal. Acc.* balanced accuracy; *CVC* cross-validation consistency; *OR* odds ratio; *95% CI* 95% confidence interval

Bold values indicate statistical significance

*p* values were obtained using χ2 tests

### FPRP analysis

FPRP analysis was employed to validate the reliability of the observed associations between *ESR1* and *APOE* SNPs and the risk of ONFH (Table 8). The associations, reflected by FPRP values below 0.2, are notable findings of significance. Significantly, in both the overall analysis and subgroup analyses based on age (> 51 years), females, and non-smoking status, rs9322332 demonstrated a noteworthy association with a reduced risk of ONFH, particularly at a prior probability of 0.25. With a prior probability of 0.1, the association between rs9322332 and a lowered risk of ONFH remained significant in the overall analysis (A vs C: FPRP = 0.167, power = 0.983; AA vs CC: FPRP = 0.085, power = 0.600; CA + AA vs CC: FPRP = 0.070, power = 0.648; Additive: FPRP = 0.103, power = 0.964), as well as in stratified analyses based on age (> 51) (AA vs CC: FPRP = 0.084, power = 0.107; CA + AA vs CC: FPRP = 0.043, power = 0.079; Additive: FPRP = 0.021, power = 0.423) and females (AA vs CC: FPRP = 0.136, power = 0.108; CA + AA vs CC: FPRP = 0.149, power = 0.173). Even with a prior probability of 0.01, rs9322332 continued to exhibit an association with a reduced risk of ONFH among individuals older than 51 years (Additive: FPRP = 0.188, power = 0.423) under the additive model. Based on the results of the FPRP analysis, this study provided further evidence for a strong association between *ESR1*-rs9322332 and a reduced risk of ONFH.

**Table 8:** Results of FPRP analysis for significant findings

Genotype	Crude OR (95% CI)	*p*	Statistical power	Prior probability				
				0.25	0.1	0.01	0.001	0.0001
rs9322332 (A < C)								
Overall analysis								
A vs C	0.81 (0.68–0.97)	0.020	0.983	**0.063**	**0.167**	0.689	0.957	0.996
AA vs CC	0.69 (0.53–0.90)	0.006	0.600	**0.030**	**0.085**	0.505	0.912	0.990
CA + AA vs CC	0.70 (0.54–0.90)	0.006	0.648	**0.024**	**0.070**	0.452	0.893	0.988
Additive	0.79 (0.66–0.95)	0.013	0.964	**0.037**	**0.103**	0.557	0.927	0.992
Age˃51								
CA vs CC	0.47 (0.28–0.80)	0.005	0.099	**0.141**	0.330	0.844	0.982	0.998
AA vs CC	0.52 (0.35–0.77)	0.001	0.107	**0.030**	**0.084**	0.502	0.911	0.990
CA + AA vs CC	0.51 (0.35–0.74)	*p* < 0.001	0.079	**0.015**	**0.043**	0.329	0.832	0.980
Additive	0.65 (0.50–0.84)	0.001	0.423	**0.007**	**0.021**	**0.188**	0.701	0.959
Female								
AA vs CC	0.51 (0.34–0.78)	0.002	0.108	**0.050**	**0.136**	0.634	0.946	0.994
CA + AA vs CC	0.55 (0.37–0.82)	0.004	0.173	**0.055**	**0.149**	0.658	0.951	0.995
Smoking-No								
CA + AA vs CC	0.68 (0.47–0.99)	0.042	0.541	**0.197**	0.424	0.890	0.988	0.999
Additive	0.77 (0.59–0.99)	0.042	0.869	**0.125**	0.301	0.825	0.979	0.998

*OR* odds ratio; *95% CI* 95% confidence interval

While the false-positive report probability threshold at 0.2, noteworthy findings are presented

Bold values indicate noteworthy findings

## Discussion

In the present study, we investigated the potential link between *ESR1* and *APOE* gene polymorphisms and the risk of ONFH. Our results suggested a significant association between *ESR1*-rs9322332 and a significantly decreased risk of ONFH under the homozygous (AA vs.CC: OR = 0.69, 95% CI [0.53–0.90], *p* = 0.006), dominant (CA + AA vs. CC: OR = 0.70, 95% CI [0.54–0.90], *p* = 0.006), and additive (OR = 0.79, 95% CI [0.66–0.95], *p* = 0.013) models. The stratified analysis revealed that this polymorphism has a protective effect against ONFH in non-smoker and aged over 51 years old. These results emphasized the significance of *ESR1*-rs9322332 in the pathogenesis and advancement of ONFH and suggest its potential as a novel biomarker for ONFH treatment.

Regarding the *ESR1* rs2982573, rs10872678, and rs9322332, currently, there is limited research available. Liu et al. conducted a study showing that individuals in the Taiwanese population carrying the TC + CC genotypes of *ESR1* rs2982573 had a lower likelihood of developing osteoporosis when consuming at least three cups of coffee per week [19]. However, our study did not observe any relationship between *ESR1* rs2982573 and the risk of ONFH. This discrepancy could be attributed to differences in the geographical locations of the study participants, as our study focused on individuals from inland China. For rs9322332, Andrew May et al. discovered that carrying the C allele of *ESR1*-rs9322332 was associated with a decrease in bone mineral content among black South African children [20]. Interestingly, our study revealed a correlation between carrying the A allele of *ESR1*-rs9322332 and a lowered risk of ONFH, indicating a potential association between the rs9322332 mutation’s impact on bone mineral content and the reduced risk of ONFH. However, there are no studies on the association between rs10872678 and ONFH or other diseases.

Regarding the *APOE* rs7259620 and rs769446, there has been a significant amount of research conducted thus far. Cai et al. investigated the correlation between *APOE* gene polymorphisms, diet, and dyslipidemia in the Yao minority area, and found no significant association between rs7259620 and dyslipidemia [21]. Park et al. conducted a genetic variation selection study associated with the risk of hyper-LDL-cholesterolemia and found that rs7259620 is associated with a reduced risk of hyper-LDL-cholesterolemia [22]. Furthermore, it has been reported that rs7259620 is associated with both Alzheimer’s disease and coronary heart disease [23, 24]. In regards to rs769446, Ereqat et al. investigated the impact of *APOE* gene variations on the risk of dyslipidemia in diabetes, and no statistical differences were observed in rs449647 variants among T2D patients with and without dyslipidemia [25]. Moreover, multiple studies have also explored the association of rs7259620 with Alzheimer’s disease and coronary heart disease, but no significant association was observed [26–29]. In our results, we explored the association between two SNPs of the *APOE* gene and the risk of ONFH, but no significant association was found between them. We speculate that such results may be related to the study participants’ region of residence, race, type of disease, and a variety of other factors.

The onset of ONFH is affected by many factors, including age, gender, and smoking status. ONFH is a disabling condition that primarily affects young to middle-aged individuals. In China, the mean age at diagnosis is 50.40 ± 13.71 years, with a predominant number of patients falling within the age range of 41 to 60 years old [30]. Additionally, epidemiological data indicates that the incidence of ONFH is higher in males compared to females [30, 31]. Considering the significance of age and gender as risk factors for ONFH, we conducted a stratified analysis to explore the influence of *ESR1* and *APOE* gene polymorphisms on the risk of developing ONFH. Our findings indicated that the *ESR1*-rs9322332 polymorphism was linked to a decreased risk of ONFH among individuals aged over 51 years, suggesting an age-dependent effect of *ESR1*-rs9322332 on ONFH risk. We also found a significant correlation between *ESR1*-rs9322332 polymorphism and a reduction in ONFH risk among female participants. However, considering that the HWE *p* value < 0.05 in the female population (Additional file 1: Table S3), further validation is needed to determine whether the correlation between *ESR1*-rs9322332 and ONFH risk depends on gender. In addition, previous research has also demonstrated a positive association between smoking and an elevated risk of ONFH. As demonstrated by the study conducted by Takahashi et al., current smokers, individuals with a smoking consumption exceeding 20 cigarettes per day, and those with 26 pack-years or more, have ONFH risks that are 3.89 (95% CI 1.46–10.4), 3.89 (95% CI 1.22–12.4), and 4.26 (95% CI 1.32–13.7) times higher, respectively, compared to non-smokers [32]. From this observation, it is evident that the more one smokes, the greater the risk of developing ONFH. These findings emphasize the significance of taking behavioral habits into account when investigating the relationship between genetic factors and the risk of ONFH.

Certain limitations exist in this study that should be acknowledged. Firstly, the participants were exclusively recruited from a single hospital, possibly leading to selection bias. Secondly, functional experiments were not performed in this study. In future studies, we will expand the sample size to further investigate the association between *ESR1* rs9322332 and the risk of ONFH, and validate our findings through in vivo animal experiments.

## Conclusions

This study has established a strong link between *ESR1*-rs9322332 and a lower incidence of ONFH, particularly among individuals over the age of 51 and non-smokers. However, further validation with a larger sample size is necessary. In summary, this study provides valuable insights into the role of *ESR1* gene polymorphisms in the prevention and diagnosis of ONFH.

### Supplementary Information

**Additional file 1**. **Table S1**. The primers of the selected SNPs. **Table S2**. Association between *ESR1* and *APOE* polymorphisms and ONFH risk stratified by clinical staging. **Table S3**. *P* values obtained through Hardy-Weinberg equilibrium under overall and subgroup stratification.

## Data Availability

The data that support our findings are available from the corresponding author upon reasonable requirements.

## Abbreviations

ONFH: Osteonecrosis of the femoral head
ORs: Odds ratios
Cis: Confidence intervals
THA: Total hip arthroplasty
BMCTs: Bone marrow-derived cell therapies
CD: Core decompression
ER: Estrogen receptor
ESR1: Estrogen Receptor 1
APOE: Apolipoprotein E
VLDL: Very low-density lipoproteins
HDL: High-density lipoproteins
CT: Computed tomography
MRI: Magnetic resonance imaging
HWE: Hardy–Weinberg equilibrium
MDR: Multifactor dimensionality reduction
FPRP: False positive report probability
