# Supplementary material for: Correlation between ESR1 and APOE gene polymorphisms and risk of osteonecrosis of the femoral head: a case–control study
Source: J Orthop Surg Res. 2023 Dec 15;18:968. doi: 10.1186/s13018-023-04447-4 (PMC10722694; doi:10.1186/s13018-023-04447-4)
Supplement: Supplementary file 1 — Additional file 1. Table S1. The primers of the selected SNPs. Table S2. Association between ESR1 and APOE polymorphisms and ONFH risk stratified by clinical staging. Table S3. P values obtained through Hardy-Weinberg equilibrium under overall and subgroup stratification. [file 13018_2023_4447_MOESM1_ESM.docx]

**Table S1** The primers of the selected SNPs

| **SNP_ID** | **1st-PCRP** | **2nd-PCRP** | **UEP_SEQ** | **Director** |
| --- | --- | --- | --- | --- |
| **rs2982573** | ACGTTGGATGCAGAATACATAGAGCAGGAC | ACGTTGGATGCCTTGTGTAAGTGAATCTTG | AGTGAATCTTGTTGAGATTTTATG | F |
| **rs10872678** | ACGTTGGATGGCCTTCAACTTTGTTCTCCC | ACGTTGGATGGGGAGTGTCATGGTAAGAGA | ggAGGTAGAGCCACAAATAG | R |
| **rs9322332** | ACGTTGGATGGTCTTGTATACGCAGACCAG | ACGTTGGATGCTGAAACCAAGTGGAATTGC | gggaCAAGTGGAATTGCATTTTTATA | R |
| **rs7259620** | ACGTTGGATGAATGAGTCCCAGTCTCTCCC | ACGTTGGATGTTTCAGAGGAGAAACCCGTG | GTGGTTCAGCAGCAAGA | R |
| **rs769446** | ACGTTGGATGGAAAGAAAGTAGGGCTAGGG | ACGTTGGATGCTTAAGTGATTCGCCCACTG | ttacGGGATTACAGGCGTGAGC | F |

SNP: single nucleotide polymorphism; PCRP: polymerase chain reaction primer; UEP: unextended primer; SEQ: sequence.

**Table S2** Association between *ESR1* and *APOE* polymorphisms and ONFH risk stratified by clinical staging

| **SNP_ID** | **Model** | **Genotypes** | **Stage III-IV** | **Stage I-II** | **OR (95% CI)** | ***p*** |
| --- | --- | --- | --- | --- | --- | --- |
| rs2982573 | Co-dominant | TT | 146 | 50 | 1 |  |
|  |  | TC | 48 | 18 | 1.28 (0.14-11.78) | 0.828 |
|  |  | CC | 5 | 1 | 0.88 (0.44-1.75) | 0.720 |
|  | Dominant | TT | 146 | 50 | 1 |  |
|  |  | TC+CC | 53 | 19 | 0.91 (0.47-1.77) | 0.775 |
|  | Recessive | TT+TC | 194 | 68 | 1 |  |
|  |  | CC | 5 | 1 | 1.32 (0.14-12.07) | 0.806 |
|  | Additive | — | — | — | 0.95 (0.52-1.71) | 0.856 |
| rs10872678 | Co-dominant | TT | 123 | 35 | 1 |  |
|  |  | TC | 64 | 31 | 0.70 (0.17-2.78) | 0.609 |
|  |  | CC | 12 | 3 | 0.55 (0.29-1.02) | 0.059 |
|  | Dominant | TT | 123 | 35 | 1 |  |
|  |  | TC+CC | 76 | 34 | 0.56 (0.31-1.03) | 0.063 |
|  | Recessive | TT+TC | 187 | 66 | 1 |  |
|  |  | CC | 12 | 3 | 0.89 (0.23-3.46) | 0.870 |
|  | Additive | — | — | — | 0.67 (0.41-1.09) | 0.106 |
| rs9322332 | Co-dominant | CC | 84 | 24 | 1 |  |
|  |  | CA | 96 | 34 | 0.40 (0.15-1.06) | 0.066 |
|  |  | AA | 19 | 11 | 0.77 (0.40-1.47) | 0.425 |
|  | Dominant | CC | 84 | 24 | 1 |  |
|  |  | CA+AA | 115 | 45 | 0.68 (0.36-1.26) | 0.217 |
|  | Recessive | CC+CA | 180 | 58 | 1 |  |
|  |  | AA | 19 | 11 | 0.46 (0.19-1.14) | 0.094 |
|  | Additive | — | — | — | 0.67 (0.42-1.06) | 0.084 |
| rs7259620 | Co-dominant | GG | 104 | 34 | 1 |  |
|  |  | GA | 81 | 28 | 0.73 (0.24-2.20) | 0.577 |
|  |  | AA | 14 | 6 | 1.05 (0.56-1.99) | 0.877 |
|  | Dominant | GG | 104 | 34 | 1 |  |
|  |  | GA+AA | 95 | 34 | 0.99 (0.54-1.81) | 0.969 |
|  | Recessive | GG+GA | 185 | 62 | 1 |  |
|  |  | AA | 14 | 6 | 0.72 (0.25-2.07) | 0.537 |
|  | Additive | — | — | — | 0.93 (0.58-1.49) | 0.770 |
| rs769446 | Co-dominant | TT | 168 | 59 | 1 |  |
|  |  | TC | 30 | 10 | — | 0.999 |
|  |  | CC | 1 | 0 | 0.82 (0.35-1.92) | 0.649 |
|  | Dominant | TT | 168 | 59 | 1 |  |
|  |  | TC+CC | 31 | 10 | 0.87 (0.37-2.01) | 0.741 |
|  | Recessive | TT+TC | 198 | 69 | 1 |  |
|  |  | CC | 1 | 0 | — | 0.999 |
|  | Additive | — | — | — | 0.93 (0.42-2.07) | 0.854 |

ONFH: Osteonecrosis of the femoral head; SNP: single nucleotide polymorphism; OR: odds ratio; 95% CI: 95% confidence interval.

*p*-value was calculated by logistic regression analysis with adjustments for age and gender.

Bold values indicate statistical significance.

**Table S3.** Hardy-Weinberg equilibrium *p*-value in controls under overall stratification and subgroup stratification.

| **SNP_ID** | **Overall** | **Males** | **Females** | **Age ˃ 51** | **Age ≤ 51** | **Smoking-Yes** | **Smoking-No** | **Clinical staging** |
| --- | --- | --- | --- | --- | --- | --- | --- | --- |
| **rs2982573** | 0.738 | 0.379 | 0.120 | 1.000 | 0.797 | 1.000 | 0.809 | 1.000 |
| **rs10872678** | 0.195 | 1.000 | **0.036** | 0.451 | 0.374 | 0.119 | 1.000 | 0.358 |
| **rs9322332** | 0.057 | 0.641 | **0.015** | 0.124 | 0.196 | **0.047** | 0.510 | 1.000 |
| **rs7259620** | 0.524 | 0.667 | 0.637 | 0.461 | 1.000 | 0.480 | 1.000 | 1.000 |
| **rs769446** | 0.607 | 0.150 | 0.465 | 1.000 | 0.709 | 0.151 | 0.450 | 1.000 |

*p* > 0.05 indicated that genotype distribution of each locus met HWE.
